# Cognitive impairment in the schizophrenia spectrum: exploring the relationships of the g-factor with sociodemography, psychopathology, neurodevelopment, and genetics

**DOI:** 10.1017/S0033291724002538

**Published:** 2024-11

**Authors:** Delphine Yeh, Qin He, Emma Krebs, Anton Iftimovici, Gilles Martinez, Julie Bourgin-Duchesnay, Fayçal Mouaffak, Charlotte Danset-Alexandre, Marie de Gasquet, Célia Jantac, Narjes Bendjemaa, Boris Chaumette, Marie-Odile Krebs, Linda Scoriels

**Affiliations:** 1 Laboratoire de Physiopathologie des Maladies Psychiatriques, Institute of Psychiatry and Neuroscience of Paris, INSERM U1266, Université Paris Cité, F-75014 Paris, France; 2 Laboratoire Mémoire, Cerveau et Cognition, Université Paris Cité, F-92100 Boulogne-Billancourt, France; 3 GHU Paris Psychiatrie et Neurosciences, Hôpital Sainte Anne, F-75014 Paris, France; 4 Groupe Hospitalier Nord Essonne, Hôpital Paris Saclay, F-91400 Orsay, France; 5 Centre for Epidemiology and Population Health, INSERM U1018, Équipe Psychiatrie du Développement et Trajectoires ‘DevPsy’, Université Paris-Saclay, F-94807 Villejuif, France; 6 Établissement Public de Santé Ville Evrard, Pôle 93G04, F-93200 Saint-Denis, France; 7 Centre Hospitalier d'Erstein, Pôle ISIS, Équipe d'Appui au Rétablissement, F-67150 Erstein, France; 8 Department of Psychiatry, McGill University, Montreal, Canada; 9 Laboratoire de Psychologie du Développement et de l’Éducation de l'Enfant, CNRS, Université Paris Cité, F-75005 Paris, France

**Keywords:** clinical high-risk, cognition, functional outcomes, genetic risk, G-factor, neurodevelopmental load, neuropsychology, prodrome, psychosis, schizophrenia

## Abstract

**Background:**

Cognitive impairment constitutes a prevailing issue in the schizophrenia spectrum, severely impacting patients' functional outcomes. A global cognitive score, sensitive to the stages of the spectrum, would benefit the exploration of potential factors involved in the cognitive decline.

**Methods:**

First, we performed principal component analysis on cognitive scores from 768 individuals across the schizophrenia spectrum, including first-degree relatives of patients, individuals at ultra-high risk, who had a first-episode psychosis, and chronic schizophrenia patients, alongside 124 healthy controls. The analysis provided 10 g-factors as global cognitive scores, validated through correlations with intelligence quotient and assessed for their sensitivity to the stages on the spectrum using analyses of variance. Second, using the g-factors, we explored potential mechanisms underlying cognitive impairment in the schizophrenia spectrum using correlations with sociodemographic, clinical, and developmental data, and linear regressions with genotypic data, pooled through meta-analyses.

**Results:**

The g-factors were highly correlated with intelligence quotient and with each other, confirming their validity. They presented significant differences between subgroups along the schizophrenia spectrum. They were positively correlated with educational attainment and the polygenic risk score (PRS) for cognitive performance, and negatively correlated with general psychopathology of schizophrenia, neurodevelopmental load, and the PRS for schizophrenia.

**Conclusions:**

The g-factors appeared as valid estimators of global cognition, enabling discerning cognitive states within the schizophrenia spectrum. Educational attainment and genetics related to cognitive performance may have a positive influence on cognitive functioning, while general psychopathology of schizophrenia, neurodevelopmental load, and genetic liability to schizophrenia may have an adverse impact.

## Introduction

Schizophrenia is a chronic psychiatric disorder affecting approximately 1% of the global population, reducing lifespan by 20 years (Kahn et al., 2015). It involves generalized cognitive deficits across multiple domains (Dickinson, Ragland, Gold, & Gur, 2008; Fioravanti, Bianchi, & Cinti, 2012; Le Roy, 2011), severely impairing functional outcomes (McCutcheon, Keefe, & McGuire, 2023; Prouteau & Verdoux, 2011). Cognitive deficits are present, although milder, at prodromal stages of the schizophrenia spectrum, i.e. in individuals at ‘ultra-high risk’ (UHR) of psychosis (Fusar-Poli et al., 2012, 2013). Around 25% of UHR convert to chronic psychosis within 3 years of follow-up (Salazar de Pablo et al., 2021), and these converters exhibit more severe baseline cognitive deficits than non-converters (Fusar-Poli et al., 2013). Unaffected first-degree relatives (FDR) also present poorer cognition than healthy controls (HC) with no family history of schizophrenia (Snitz, MacDonald, & Carter, 2006). Understanding the mechanisms underlying cognitive deficits is essential for early intervention and prevention of disorder progression. However, cognitive assessments are time-consuming and use varied tests, hindering large cohort collection and cross-study comparisons.

To address this issue, the general factor of intelligence, or *g-factor*, appeared as a relevant construct. The g-factor measures overall cognitive ability, capturing common core features of cognitive ability across different cognitive tasks (Jensen, 1998; Spearman, 1904). It is considered as the single higher-order factor at the apex of the hierarchical intelligence models built upon the Cattell–Horn–Carroll theory of intelligence (Carroll, 1993, 2003; Cattell & Horn, 1978; Schneider & McGrew, 2012). Computationally, the g-factor can be defined as the first component of a principal component analysis (PCA) performed on an individual's cognitive test scores (Jensen, 1998). The g-factors derived from different test batteries have been demonstrated to be highly correlated (Johnson, Bouchard, Krueger, McGue, & Gottesman, 2004, 2008), supporting the existence of a single global intelligence factor and the consistency of its computation, provided the test batteries assess sufficiently diverse cognitive abilities (Dickinson, Goldberg, Gold, Elvevåg, & Weinberger, 2011). The g-factor thus enables comparisons and data pooling across studies with different cognitive assessments, a major stake in the era of big data.

Using a specifically selected neuropsychological battery, the g-factor has been validated as a higher-order cognitive factor in schizophrenia patients (SZ), their unaffected siblings, and HC (Dickinson et al., 2011). However, further investigation is required to ascertain its generalizability to other samples and test batteries, and its potential in discriminating subgroups along the schizophrenia spectrum. The literature would also benefit from a more comprehensive understanding of the sociodemographic, clinical, and genetic contributions to cognition, which could, in turn, inform the development of alternative treatments. Indeed, antipsychotic medication, the primary treatment for psychosis, appeared relatively ineffective in reducing cognitive deficits (Keefe et al., 2007; Millan et al., 2016).

Cognitive phenotypes have a high heritability (Blokland et al., 2017; Mallet, Le Strat, Dubertret, & Gorwood, 2020). Polygenic risk scores (PRS) estimate the genetic heritability of traits based on genome-wide association studies (GWAS) (Choi, Mak, & O'Reilly, 2020). Previous research by our team and others found significant correlations between cognition and several PRS in UHR patients (He et al., 2021) and within SZ cases (Richards et al., 2019), but lacked comparisons with HC and the full schizophrenia spectrum.

The present study had three objectives: first, to compute and validate g-factors derived from different cognitive test batteries as global cognitive scores that can discriminate between the stages of the schizophrenia spectrum; second, to use the g-factors to investigate potential mechanisms underlying cognitive impairment in the schizophrenia spectrum; and third, to determine which test battery produced the most informative g-factor for discriminating schizophrenia spectrum subgroups and correlating with other dimensions. PCA was used to derive 10 g-factors, the correlations of which with intelligence quotient and with one another were examined for validation as global cognitive scores. Analysis of variance (ANOVA) assessed differences across subgroups, namely HC, FDR, UHR, first-episode psychosis (FEP), and SZ. Correlations between the g-factors, sociodemographic, and clinical data were investigated. Linear regressions between the g-factors and the PRS for cognitive performance and for schizophrenia were conducted, and results pooled through meta-analyses to establish global correlations between PRS and cognition.

## Materials and methods

### Participants

The present retrospective study compiled data from 892 individuals included in six previous studies coordinated by M.-O. K. at GHU Paris Psychiatry and Neuroscience, assessing various aspects, including the influence of genetics or environmental factors in psychosis and its transition. Baseline visits were selected for interventional and longitudinal studies to prevent bias. Data were collected between 2005 and 2021. Further details on the individual studies can be found in previous publications (Gay et al., 2013; Krebs et al., 2014; Magaud et al., 2014; Martinez et al., 2017). Study abbreviations are detailed in the ‘Acknowledgments’ section.

Inclusion criteria for the present analyses were an age over 15 years old, and diagnoses within the schizophrenia spectrum: 338 UHR identified using the Comprehensive Assessment of At-Risk Mental State (Krebs et al., 2014) (ICAAR, START, PrEPP studies), 100 FEP (ICAAR and PRIMEPI studies), and 167 SZ diagnosed with DSM-IV criteria after the Diagnosis Interview for Genetic Studies 3.0 (Nurnberger et al., 1994) (AUSZ, PrEPP, PRIMEPI, and PsyDEV studies). Additionally, 124 HC with no personal or familial psychiatric history up to second-degree relatives were included (AUSZ, START, and PsyDEV studies), and 163 FDR (PsyDEV study). Exclusion criteria included severe somatic or neurological disorders, intelligence quotient below 70, severe substance use disorders for more than 5 years, or involuntary hospitalization. Free and informed written consent was obtained from all participants or their legal representatives. The studies adhered to the French regulatory framework and received ethics committees approvals.

### Cognitive data

The French version of the Wechsler Adult Intelligence Scale (WAIS) was administered in four studies: PrEPP, ICAAR, and AUSZ studies used the 3rd edition (WAIS-III) (Wechsler, 1997) with 199 UHR, 35 FEP, 53 SZ, and 25 HC; START study used the 4th edition (WAIS-IV) (Wechsler, 2008) with 44 UHR and 7 HC. The WAIS is commonly used to derive the g-factor, hence our focus on this scale and its subtests.

Due to time constraints in larger studies, a shorter cognitive assessment, referred to as ‘Minicog’, was used in five studies to reduce participant burden. This assessment takes approximately 30 min, compared to 90 min for the WAIS. The ICAAR and AUSZ studies used the complete Minicog (‘Minicog 6’) with 106 UHR, 29 FEP, 34 SZ, and 21 HC. The ICAAR, AUSZ, START, PsyDEV, and PRIMEPI studies used the partial Minicog (‘Minicog 3’) with 157 UHR, 77 FEP, 93 SZ, 43 HC, and 92 FDR. The Minicog comprised: Verbal Fluency Test (Cardebat, Doyon, Puel, Goulet, & Joanette, 1990); Trail Making Test A & B (Reitan, 1956); Proverbs, i.e. item N5 from the Positive and Negative Syndrome Scale (PANSS) (Lançon, Reine, Llorca, & Auquier, 1999); WAIS Similarities; Wechsler Memory Scale-IV Coupled Words subtest (Wechsler, 2010); and the French adaptation of the National Adult Reading Test (Mackinnon, Ritchie, & Mulligan, 1999). The partial Minicog included the first three of these tests.

### Computation of the g-factors

The g-factors were derived from 10 combinations of neuropsychological tests. Seven g-factors were derived from the WAIS: *g WAIS global* was derived from the Verbal and Performance intelligence quotients of the WAIS-III (*n* = 323 individuals); *g WAIS indices* was derived from the four indices of the WAIS-IV, i.e. Verbal Comprehension, Working Memory, Perceptual Reasoning, and Processing Speed (*n* = 51); *g WAIS-III* was derived from the nine main subtests of the WAIS-III (*n* = 312); *g WAIS-IV* was derived from the 10 main subtests of the WAIS-IV (*n* = 51); *g WAIS 8* was derived from the eight subtests that were common to both WAIS-III and WAIS-IV (*n* = 363); *g WAIS 4* was derived from four subtests representing each index of the WAIS-IV (Gignac, 2015), i.e. Similarities, Digit Span, Matrix Reasoning, and Symbol Search (*n* = 51); and *g WAIS 2* was derived from Coding and Information, identified as the most valid and reliable dyad for estimating the Full-Scale Intelligence Quotient (FSIQ) (Girard, Axelrod, Patel, & Crawford, 2015) (*n* = 366). It is important to note the distinction between the g-factor and FSIQ, although the WAIS is based on the Cattell–Horn–Carroll theory, as are most modern intelligence tests. The FSIQ is a composite score derived from standardized intelligence subtests measuring an individual's overall cognitive ability compared to a normative sample, whereas the g-factor is a theoretical construct derived from statistical analysis and representing underlying cognitive ability across various cognitive tasks. Two g-factors were derived from the Minicog: *g Minicog 6* derived from the complete Minicog (*n* = 190) and *g Minicog 3* derived from the partial Minicog (*n* = 462). Finally, one g-factor was computed by pooling data from the WAIS and the Minicog, using WAIS FSIQ and the scores of the complete Minicog, excluding WAIS Similarities to avoid redundancy (*n* = 239). In total, at least one g-factor could be computed for 598 individuals.

### Whole-genome genotyping data

Both cognitive and genotyping data were available for 220 individuals (40 HC, 104 UHR, 5 FEP, 71 SZ). Despite the small sample size for FEP, their data were retained as our objective was to investigate the relationship between cognition and genetics across the schizophrenia spectrum as a whole, rather than within each subgroup. Genotyping was conducted using the high-throughput genome-wide Illumina Infinium PsychArray-24 v1.3 BeadChip (Illumina, California, USA). Summary statistics from recent GWAS related to cognitive performance (Lee et al., 2018) and schizophrenia (Schizophrenia Working Group of the PGC et al., 2022) were used as base data.

### Computation of the PRS

Quality control on raw genotyping data was performed using *PLINK* (v1.9) (Chang et al., 2015), following standard procedures (Choi et al., 2020; He et al., 2021; Marees et al., 2018). Single-nucleotide polymorphisms (SNP) with a missingness rate exceeding 2% and Hardy–Weinberg equilibrium *p* values below 10^–6^ were removed. Pruning retained only independent SNP. A sex check excluded one SZ patient with inconsistent gender assignment. Imputation was performed with the Sanger Imputation Service (McCarthy et al., 2008) to enhance statistical power (He et al., 2021; Porcu, Sanna, Fuchsberger, & Fritsche, 2013), using EAGLE2 for pre-phasing and PBWT for imputation on a reference panel combining 1000 Genomes Phase 3 and UK10K. Imputed SNP with information scores below 0.8, missingness rates above 2%, and deviations from Hardy–Weinberg equilibrium were filtered out. The remaining SNP were pruned. Relatedness analysis excluded five FDR or second-degree relatives, using a cut-off coefficient of 0.125. Population genetic stratification was assessed with a PCA using *Peddy* (Pedersen & Quinlan, 2017) and excluded 19 ethnic outliers, retaining only individuals of European ancestry.

Following quality control, 195 individuals remained (38 HC, 88 UHR – of whom 85 were also included in a previous analysis from our team focused on UHR only [He et al., 2021] – 5 FEP, 64 SZ). A total of 131 individuals had a *g WAIS global*, 130 a *g WAIS-III*, 143 a *g WAIS 8*, 144 a *g WAIS 2*, 109 a *g Minicog 6*, 178 a *g Minicog 3*, and 127 a *g Mix*. Thirteen individuals had a *g WAIS indices*, a *g WAIS-IV*, and a *g WAIS 4*, but those samples were excluded from PRS calculations due to the small sample size.

PRS were computed using *PRSice-2* (v2.3.3) (Choi & O'Reilly, 2019), filtering out SNP with information scores below 0.8 or minor allele frequencies below 5%. Analyses were conducted for each PRS and the seven g-factor samples at 14 *p* value thresholds (He et al., 2021), retaining scores computed at the most predictive threshold for each PRS.

### Psychopathological and neurodevelopmental data

Patients completed the PANSS (Kay, Fiszbein, & Opler, 1987; Lançon et al., 1999) that assessed positive and negative symptoms, and general psychopathology; the Brief Psychiatric Rating Scale-Expanded (BPRS-E) (Dingemans, Linszen, Lenior, & Smeets, 1995; Mouaffak et al., 2010) that assessed general psychiatric symptoms; the Developmental Disorders Screening Scale (DDSS) (Martinez, 2017) that assessed developmental disorders in childhood; and the Neurological Soft Signs Examination (NSS) (Krebs, Gut-Fayand, Bourdel, Dischamp, & Olié, 2000) that assessed subtle integrative neurological anomalies.

### Statistical analyses

Statistical analyses were conducted using R (R Core Team, 2022) with a significance level of 0.05. Descriptive statistics, one-way ANOVA, and pairwise comparisons were performed on sociodemographic, cognitive, and clinical data using the *compareGroups* package (Subirana, Sanz, & Vila, 2014).

Bartlett's sphericity tests from the *psych* package (Revelle, 2022) were used for data redundancy analysis, and PCA on 10 neuropsychological test battery scores using *FactoMineR* (Lê, Josse, & Husson, 2008) provided the g-factors. Spearman's correlations between the g-factors and FSIQ, corrected for multiple comparisons using Benjamini–Hochberg (BH) method (Benjamini & Hochberg, 1995), were computed using *stats* (R Core Team, 2022) and *psych* (Revelle, 2022) packages, and visualized using *corrplot* (Wei & Simko, 2021)

One-way between-subjects ANOVA assessed subgroup differences in g-factors using the *stats* package (R Core Team, 2022), with *post hoc* comparisons based on estimated marginal means from the *emmeans* package (Lenth, 2022), corrected using the BH method. Effect sizes were measured with the parameter *d_m_*, analogous to Cohen's *d* (Bögge, Colás-Blanco, & Piolino, 2022; Cohen, 1988).

Spearman's correlations between the g-factors, sociodemographic, and clinical data, corrected using the BH method, were analyzed. Linear regressions of standardized g-factors against standardized PRS were conducted using *stats* (R Core Team, 2022), adjusting for sex, age, and 10 top principal components from population stratification. All g-factors and PRS were scaled to have a mean of 0 and a standard deviation of 1 to standardize the regression estimates as *β* coefficients (Richards et al., 2019). Effect sizes were converted from *β* coefficients to correlations using *esc* (Lüdecke, 2019), and pooled for each PRS through meta-analyses based on the generic inverse variance method with a fixed-effects model, using *meta* (Balduzzi, Rücker, & Schwarzer, 2019). Between-sample heterogeneity was quantified using Higgins and Thompson's *I*^2^ statistic (Higgins & Thompson, 2002).

Graphs were created using *ggplot2* (Wickham, 2009), *ggpubr* (Kassambara, 2020), *ggsignif* (Ahlmann-Eltze & Patil, 2021), and *ggExtra* (Attali & Baker, 2022), and forest plots using *meta* (Balduzzi et al., 2019).

## Results

### Sample description

Descriptive statistics, ANOVA *p* values, and significant pairwise differences, for the sociodemographic, cognitive, and clinical data across the schizophrenia spectrum are provided in online Supplementary Table S1.

Regarding sociodemographic data, sex ratios were balanced for HC and FDR, but skewed toward males for UHR, FEP, and SZ (approximately 70% male). Mean age differed significantly between groups, reflecting the disease severity progression over time. There was also a bias for FDR, who were significantly older than other groups, given that they were mostly patients' parents. Only HC had significantly more years of education than all other groups (all *p* < 0.001).

Regarding cognitive data, most WAIS scores showed no significant difference between subgroups. Conversely, most tests in the Minicog battery presented significant differences between HC and patients, as well as between patient groups.

Regarding clinical scores, PANSS, BPRS, and NSS scores were found to worsen with disease severity. No group differences were observed for DDSS. Chlorpromazine equivalent doses significantly increased with disease severity.

### Computation of the g-factors and comparison across the schizophrenia spectrum

**Figure 1.**
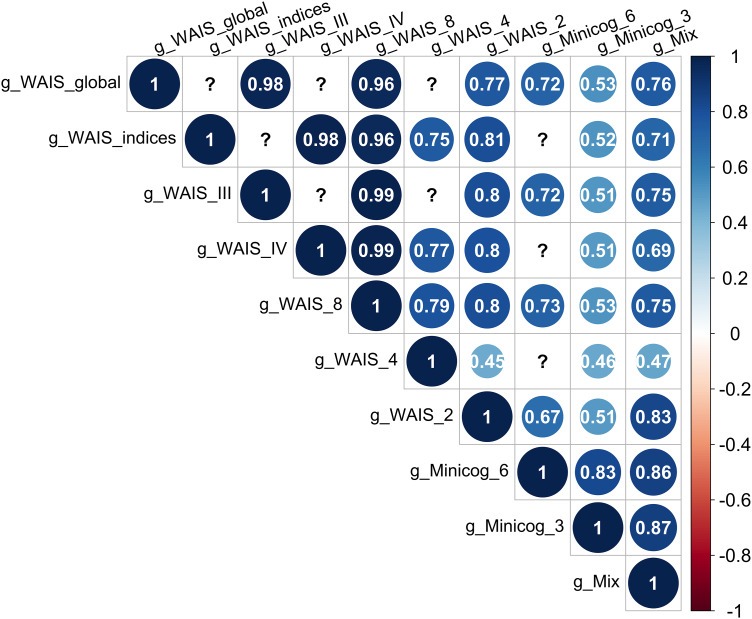
Correlations between the g-factors derived from different cognitive test combinations. All correlations are significant at a *p* value threshold of 0.05. The question marks correspond to correlations which could not be computed because the two g-factors were not available on the same sample of individuals.

Bartlett's sphericity tests were significant, justifying the use of PCA on the 10 test combinations (Table 1). The g-factors explained between 36% and 81% of the total variance in cognitive scores. Pairwise correlations between each of the g-factors and WAIS FSIQ were significant and strong (Spearman's *r* between 0.55 and 0.99), as were correlations among g-factors (*r* > 0.50 for each pairwise correlation) (Fig. 1).

**Table 1.** Combinations of cognitive tests used for the computation of 10 g-factors, proportion of total variance explained by each g-factor and its correlation with WAIS FSIQ

g-factor	*N*	*N*_HC_	*N*_FDR_	*N*_UHR_	*N*_FEP_	*N*_SZ_	Tests and scores used in the PCA	*P* (%)	*r*
g WAIS global	323	25	0	209	34	55	WAIS-III: VIQ, PIQ	81	0.99
g WAIS indices	51	7	0	44	0	0	WAIS-IV: VCI, WMI, PRI, PSI	49	0.93
g WAIS-III	312	25	0	199	35	53	WAIS-III: 9 subtests	44	0.98
g WAIS-IV	51	7	0	44	0	0	WAIS-IV: 10 subtests	36	0.92
g WAIS 8	363	32	0	243	35	53	WAIS-III and WAIS-IV: 8 common subtests	47	0.96
g WAIS 4	51	7	0	44	0	0	WAIS-IV: Similarities, Symbol Search, Digit Span, Matrix Reasoning	36	0.73
g WAIS 2	366	32	0	243	35	56	WAIS-III and WAIS-IV: Coding, Information	67	0.78
g Minicog 6	190	21	0	106	29	34	WAIS Similarities, Verbal Fluency Total, TMT B–A, fNART TIQ, Coupled Words, Proverbs	41	0.72
g Minicog 3	462	43	92	157	77	93	Verbal Fluency Total, TMT B–A, Proverbs	53	0.55
g Mix	239	21	0	150	32	36	WAIS FSIQ, Verbal Fluency Total, TMT B–A, fNART TIQ, Coupled Words, Proverbs	45	0.76

*N*, total sample size; *N*_HC_, number of healthy controls; *N*_FDR_, number of first-degree relatives; *N*_UHR_, number of patients at ultra-high risk of psychosis; *N*_FEP_, number of patients who had a first-episode psychosis; *N*_SZ_, number of patients with schizophrenia; PCA, principal component analysis; *P*: proportion of total variance explained by the g-factor; *r*, Spearman's correlation between the g-factor and WAIS (Wechsler Adult Intelligent Scale) full-scale intelligence quotient (FSIQ).

VIQ, verbal IQ; PIQ, performance IQ; VCI, Verbal Comprehension index; WMI, Working Memory index; PRI, Perceptual Reasoning index; PSI, Processing Speed index; TMT, Trail Making Test; fNART, French adaptation of the National Adult Reading Test.

**Figure 2.**
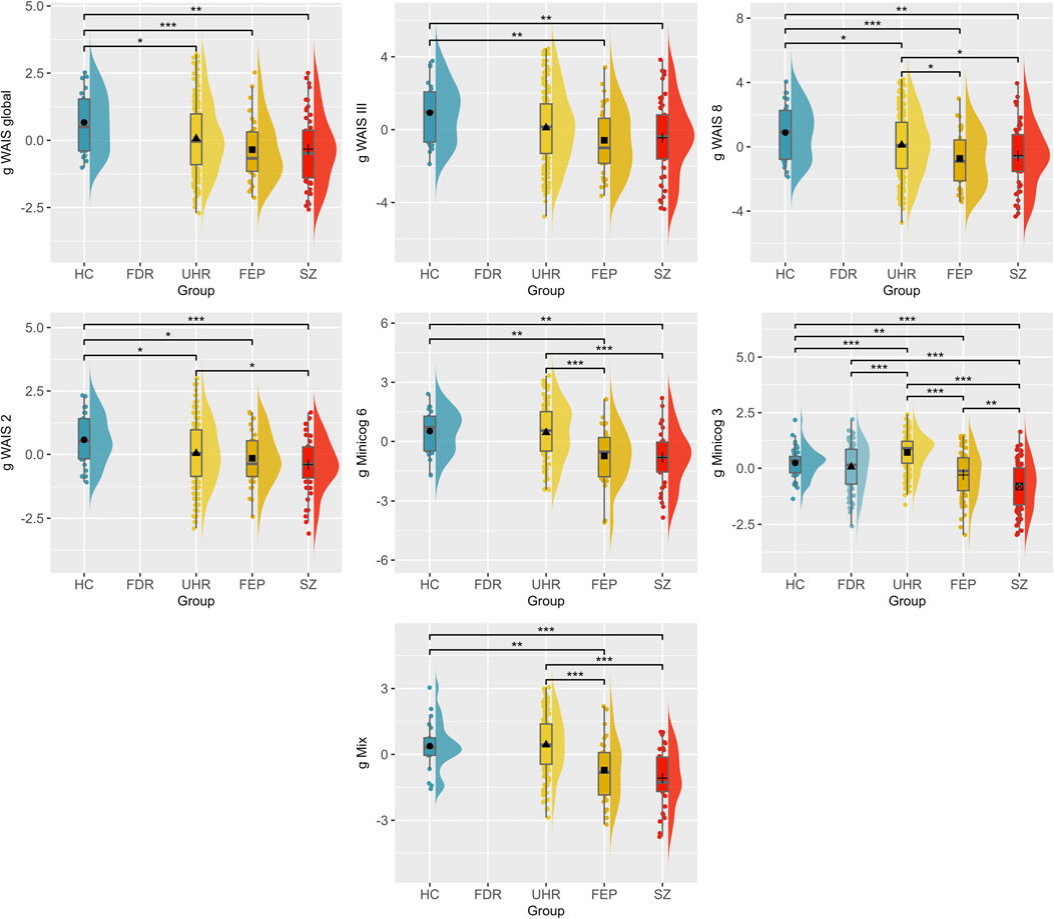
Comparison of the g-factors across the schizophrenia spectrum. HC, healthy controls; FDR, first-degree relatives; UHR, patients at ultra-high risk of psychosis; FEP, patients who had a first-episode psychosis; SZ, patients with schizophrenia. *p* value significance: *p < 0.05; **p < 0.01; ***p < 0.001.

The ANOVA performed for each g-factor revealed significant group differences for seven out of the 10 g-factors, i.e. *g WAIS global* (*F*_(3,319)_ = 4.65, *p* *=* 0.003, *η*_G_^2^ = 0.04), *g WAIS-III* (*F*_(3,308)_ = 3.99, *p* *=* 0.008, *η*_G_^2^ = 0.04), *g WAIS 8* (*F*_(3,359)_ = 5.75, *p* *=* 0.001, *η*_G_^2^ = 0.05), *g WAIS 2* (*F*_(3,362)_ = 5.37, *p* *=* 0.001, *η*_G_^2^ = 0.04), *g Minicog 6* (*F*_(3,186)_ = 10.30, *p* < 0.001, *η*_G_^2^ = 0.14), *g Minicog 3* (*F*_(4,457)_ = 39.08, *p* < 0.001, *η*_G_^2^ = 0.26), and *g Mix* (*F*_(3,235)_ = 17.98, *p* < 0.001, *η*_G_^2^ = 0.19) (Fig. 2).

All seven g-factors were significantly higher for HC compared to FEP (*g WAIS global: p* *=* 0.01, *d_m_* = 0.81; *g WAIS-III: p* *=* 0.01, *d_m_* = 0.77; *g WAIS 8: p* *=* 0.002, *d_m_* = 0.85; *g WAIS 2*: *p* *=* 0.02, *d_m_* = 0.64; *g Minicog 6: p* *=* 0.01, *d_m_* = 0.86; *g Minicog 3*: *p* *=* 0.01, *d_m_* = 0.51; *g Mix: p* *=* 0.01, *d_m_* = 0.80), and to SZ (*g WAIS global: p* *=* 0.01, *d_m_* = 0.79; *g WAIS-III: p* *=* 0.01, *d_m_* = 0.70; *g WAIS 8: p* *=* 0.002, *d_m_* = 0.76; *g WAIS 2*: *p* *=* 0.001, *d_m_* = 0.86; *g Minicog 6: p* *=* 0.001, *d_m_* = 0.98; *g Minicog 3*: *p* < 0.0001, *d_m_* = 1.15; *g Mix: p* < 0.001, *d_m_* = 1.20). Four g-factors also differed significantly between HC and UHR (*g WAIS global: p* *=* 0.048, *d_m_* = 0.48; *g WAIS 8: p* *=* 0.04, *d_m_* = 0.41; *g WAIS 2: p* *=* 0.02, *d_m_* = 0.47; *g Minicog 3*: *p* *=* 0.02, *d_m_* = −0.44). The *g Minicog 3* further differentiated FDR from UHR (*p* < 0.0001, *d_m_* = −0.69) and SZ (*p* < 0.0001, *d_m_* = 0.90), with better cognitive scores in the FDR group relative to SZ, but worse scores compared to UHR.

Regarding comparisons between patient groups, five g-factors distinguished UHR and SZ (*g WAIS 8*: *p* *=* 0.03, *d_m_* = 0.35; *g WAIS 2*: *p* *=* 0.02, *d_m_* = 0.39; *g Minicog 6*: *p* < 0.001, *d_m_* = 0.89; *g Minicog 3*: *p* < 0.0001, *d_m_* = 1.58; *g Mix*: *p* < 0.0001, *d_m_* = 1.22). Four g-factors discriminated UHR from FEP (*g WAIS 8*: *p* *=* 0.03, *d_m_* = 0.44; *g Minicog 6*: *p* *=* 0.001, *d_m_* = 0.77; *g Minicog 3*: *p* < 0.0001, *d_m_* = 0.95; *g Mix*: *p* < 0.001, *d_m_* = 0.82). Additionally, *g Minicog 3* differentiated FEP and SZ (*p* < 0.001, *d_m_* = 0.64).

All g-factors showed higher scores for HC and decreased with the more advanced stages of the disease.

### Relationships between cognition and sociodemography, psychopathology, neurodevelopmental load, medication, and genetics

No significant correlations were observed between three g-factors (*g WAIS indices*, *g WAIS-IV*, and *g WAIS 4*) and sociodemographic and clinical data. The results presented in this section will therefore focus on the seven other g-factors, i.e. *g WAIS global*, *g WAIS III*, *g WAIS 8*, *g WAIS 2*, *g Minicog 6*, *g Minicog 3*, and *g Mix* (see online Supplementary Table S2 for details).

Regarding sociodemographic data, Spearman's correlations indicated that all seven g-factors were significantly positively correlated with educational attainment. Only *g Minicog 3* was negatively correlated with age.

Regarding psychopathological data, four out of the seven g-factors (i.e. *g WAIS 2*, *g Minicog 6*, *g Minicog 3*, and *g Mix*) were negatively correlated with PANSS Total score; three g-factors were negatively correlated with PANSS Positive (i.e. *g WAIS 8*, *g Minicog 3*, and *g Mix*); three were negatively correlated with PANSS Negative (i.e. *g Minicog 6*, *g Minicog 3*, and *g Mix*), and six were negatively correlated with PANSS General Psychopathology score (all seven g-factors except *g WAIS 8*). Only *g Minicog 3* was negatively correlated with BPRS Total score.

Regarding neurodevelopmental data, six g-factors (all seven g-factors except for *g Minicog 6*) were negatively correlated with NSS Total score; one g-factor (*g Minicog 8*) was negatively correlated with NSS Motor Coordination factor; six were negatively correlated with NSS Motor Integration factor (all seven g-factors except for *g WAIS 2*); all seven g-factors were negatively correlated with NSS Sensory Integration factor; and one g-factor (*g Minicog 3*) was negatively correlated with NSS Involuntary Movements factor. No g-factor was significantly correlated with NSS Lateralization factor nor with DDSS scores.

Regarding medication, all seven g-factors were negatively correlated with chlorpromazine equivalent dose.

**Figure 3.**
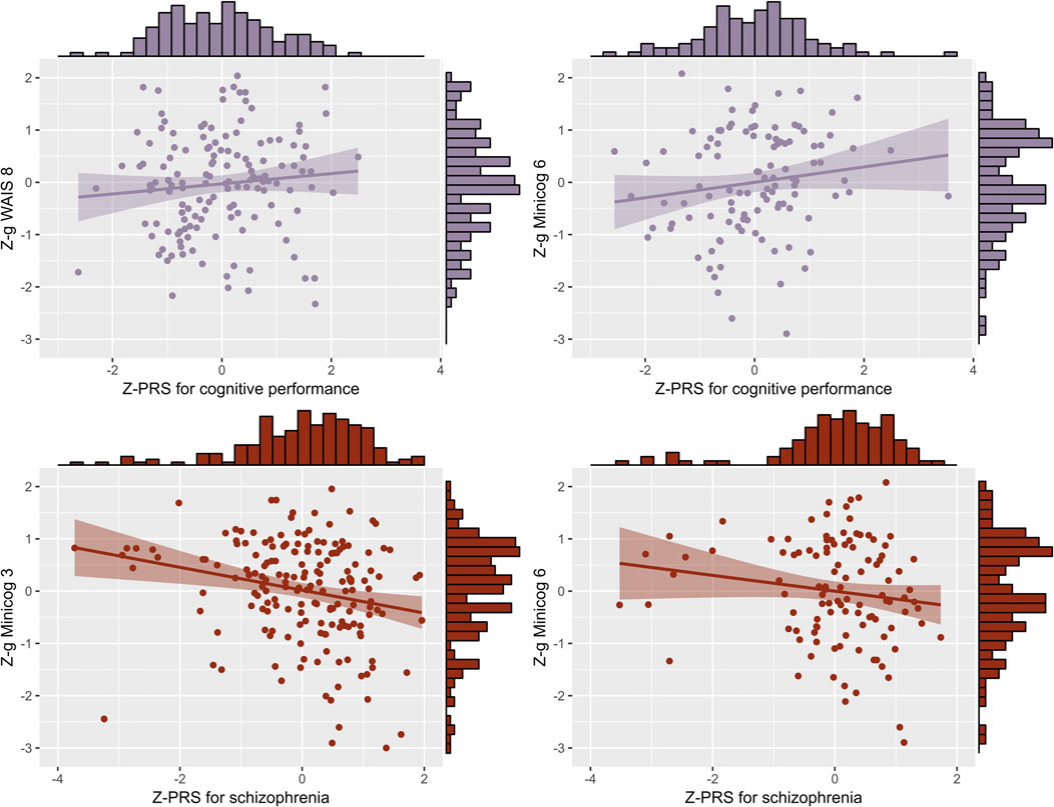
Significant linear regression models of the standardized g-factors against the standardized PRS for cognitive performance and for schizophrenia. Z-g, standardized g-factor; Z-PRS, standardized polygenic risk score.

**Figure 4.**
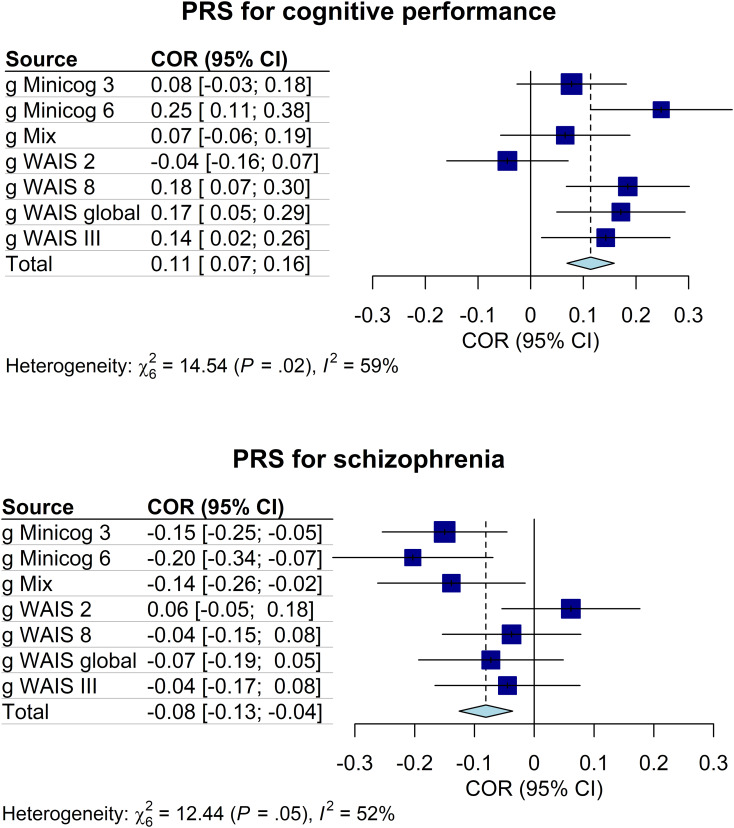
Forest plots of the correlations between the g-factors and the PRS for cognitive performance and for schizophrenia. COR, effect size as correlation; CI, confidence interval; *I*^2^, Higgins and Thompson's between-sample heterogeneity index. Error bars represent the 95% confidence intervals of the means.

Regarding genetics, linear regressions of the g-factors against the PRS indicated that the PRS for cognitive performance had a significant positive influence on *g WAIS 8* (*P*_T_ = 0.3, standardized estimate *β* = 0.19, standard error [s.e.] = 0.09, *p* *=* 0.04, proportion of g-factor variance explained by the PRS PRS.R2 = 0.03) and on *g Minicog 6* (*P*_T_ = 1 × 10^−5^, *β* = 0.25, s.e. = 0.10, *p* *=* 0.01, PRS.R2 = 0.05). The PRS for schizophrenia had a significant negative influence on *g Minicog 6* (*P*_T_ = 1 × 10^−5^, *β* = −0.20, s.e. = 0.10, *p* *=* 0.03, PRS.R2 = 0.04), and on *g Minicog 3* (*P*_T_ = 1 × 10^−5^, *β* = −0.15, s.e. = 0.07, *p* *=* 0.04, PRS.R2 = 0.02) (Fig. 3 and online Supplementary Table S3). The meta-analysis of regression results revealed a significant positive correlation between the PRS for cognitive performance and the g-factor (pooled effect size *r*_pooled_ = 0.11, *p* < 0.0001, *I*^2^ = 58.7%), and a significant negative correlation between the PRS for schizophrenia and the g-factor (*r*_pooled_ = −0.08, *p* < 0.001, *I*^2^ = 51.8%) (Fig. 4 and online Supplementary Table S4). Upon examination of the g-factors individually, those derived from WAIS scores and the complete Minicog demonstrated a stronger correlation with the PRS for cognitive performance, compared to those derived from the partial Minicog, from only two WAIS subtests, or *g Mix*. Conversely, the g-factors derived from WAIS scores exhibited weaker correlations with the PRS for schizophrenia compared to those derived from Minicog scores and *g Mix*.

## Discussion

In this study, we established and validated g-factors derived from various cognitive test batteries, providing global cognitive scores sensitive to subgroups within the schizophrenia spectrum. The g-factors were used to investigate underlying mechanisms of cognitive impairment in the spectrum. Results indicated that educational attainment and the PRS for cognitive performance were positively associated with cognition, whereas general schizophrenia psychopathology, neurodevelopmental load, medication, and the PRS for schizophrenia were negatively associated with cognitive scores across the spectrum.

The g-factors derived from 10 distinct batteries strongly correlated with FSIQ, thereby confirming their association with global intelligence (Canivez & Watkins, 2010). Despite differing methodologies, both the g-factor and FSIQ quantify general cognitive ability, serving distinct purposes in psychological assessment and research. The g-factors explained a large proportion of the variance in neuropsychological scores and were overall positively correlated with the PRS for cognitive performance, providing further evidence of their validity as global cognitive scores. Moreover, high correlations among the g-factors supported Spearman's principle of indifference of the indicator (Dickinson et al., 2011; Johnson et al., 2004, 2008; Spearman, 1904).

The following discussion will focus on seven g-factors, excluding *g WAIS indices*, *g WAIS-IV*, and *g WAIS 4*, which were only available for two unbalanced subgroups (7 HC and 44 UHR) and a limited sample size that did not pass our *post hoc* power analyses. Overall, the averaged g-factors showed a gradual decline across the schizophrenia spectrum. Three g-factors presented significantly lower scores for UHR than HC: although the effect sizes were small, they confirm that mild cognitive deficits are present at the prodromal stage (Fusar-Poli et al., 2012, 2013). All seven g-factors were lower on average in the more advanced stages of schizophrenia compared to prodromal stages, with medium-to-large effect sizes, but the difference was smaller between FEP and SZ. These findings indicate that cognitive impairment increases from prodromal to FEP stages, followed by a relative stabilization after the first episode. Notably, one g-factor, *g Minicog 3*, was on average lower for HC and FDR than UHR, potentially linked to age-related cognitive decline in the FDR, who were significantly older; other studies have also shown that some cognitive scores of FDR were more closely aligned with those of FEP than of HC (Scoriels et al., 2015). The g-factors enabled overall to discriminate between the stages of the schizophrenia spectrum when WAIS scores did not. As the g-factors capture the fundamental cognitive ability shared across tasks, they may have reduced within-group variability, thereby facilitating the detection of between-group variability. In contrast, the IQ profiles within each subgroup of the schizophrenia spectrum might have been heterogenous (Magaud et al., 2014), resulting in high within-group variability.

Correlations between the g-factors, and sociodemographic and clinical data revealed that educational attainment was associated with higher cognition, while psychopathological symptoms, neurodevelopmental load, and medication negatively affected cognitive performance. Specifically, six g-factors were negatively correlated with PANSS General Psychopathology score, whereas only one was negatively correlated with BPRS Total score, highlighting the specific relationship between cognitive functioning and schizophrenia psychopathology (Chavez-Baldini et al., 2023), but not with general psychiatric psychopathology. However, only three g-factors were correlated with PANSS Positive and Negative scores, in accordance with previous research indicating that cognitive alterations are relatively independent of schizophrenia positive and negative symptoms and constitute a distinctive dimension of the disease (Bell, Lysaker, Milstein, & Beam-Goulet, 1994; Capatina, Miclutia, & Toma, 2017; de Gracia Dominguez, Viechtbauer, Simons, van Os, & Krabbendam, 2009). Six g-factors were also negatively correlated with NSS Total score and Motor Integration factor, and seven g-factors were negatively correlated with NSS Sensory Integration factor, which is consistent with models of a neurodevelopmental origin of cognitive impairment in schizophrenia (Bora, 2015). Finally, seven g-factors were negatively correlated with chlorpromazine equivalent dose, which corroborates previous findings that higher doses of medication were associated with poorer cognition in schizophrenia (Haddad, Salameh, Sacre, Clément, & Calvet, 2023; Sweeney, Keilp, Haas, Hill, & Weiden, 1991).

The genetic contribution to cognitive impairment in the schizophrenia spectrum was also investigated. The g-factors were positively correlated with the PRS for cognitive performance and negatively correlated with the PRS for schizophrenia, when considering the entire spectrum without differentiating the subgroups, suggesting that genetic factors related to both cognitive performance and schizophrenia influence cognition in the schizophrenia spectrum. These results were congruent with those previously found by our team (He et al., 2021) and another study (Hubbard et al., 2016), with the difference that those previous studies explored correlations between FSIQ and the PRS for schizophrenia in UHR patients and in children from a population-based birth cohort, respectively. Therefore, our findings demonstrate the consistency between results based on the FSIQ and on the g-factor, and they generalize the relationship between cognition and the PRS for schizophrenia to the whole schizophrenia spectrum. Prior research has also shown that cognitive phenotypes partially share genetic etiology with schizophrenia liability (Blokland et al., 2017; Kendler, Ohlsson, Sundquist, & Sundquist, 2015; Koch, Caldwell, & Fuchs, 2013; Legge et al., 2021; Mallet et al., 2020; Mistry, Harrison, Smith, Escott-Price, & Zammit, 2018), although cognition in schizophrenia appears to be explained to a larger extent by alleles associated with cognition in the global population than risk alleles for schizophrenia *per se* (Mallet et al., 2020; Richards et al., 2019). When considered individually, the g-factors derived from WAIS scores were more correlated with the genetics linked with cognitive performance than those derived from Minicog scores and *g WAIS 2*, which was expected since the WAIS is designed primarily to evaluate global intelligence. Conversely, the g-factors derived from Minicog scores were more correlated with genetics linked to schizophrenia, which can be explained by their stronger correlations with psychopathology.

Ultimately, the g-factor derived from the partial Minicog appeared as the most informative one, as it enabled discriminating between subgroups of the schizophrenia spectrum, and was correlated with sociodemography, psychopathology, neurodevelopmental load, and schizophrenia genetics.

Several limitations should be considered. First, the sample sizes for each subgroup were relatively small, especially for genetic analyses. Second, the observed decline in the g-factor across disease stages represents an average population and does not account for within-group variability, limiting its ability to discriminate between individuals due to substantial cognitive heterogeneity. Additionally, the cross-sectional design of the study warrants longitudinal investigations to elucidate cognitive evolution across stages within individual trajectories, given the varied trajectories of cognitive development observed within the schizophrenia spectrum (Reckziegel et al., 2022). Third, the correlational nature of the analyses implied associations rather than causality, and the global approach did not discern associations between cognition and other factors within each subgroup of the spectrum separately.

Nevertheless, the study aimed to demonstrate that the g-factor could constitute a relevant indicator of the cognitive state of patients, sensitive to schizophrenia spectrum stages, and provide insights into potential mechanisms underlying cognitive impairment. Using the g-factor as a global cognitive score enabled data pooling across studies with different cognitive assessments, enhancing statistical power. Other methods have been proposed for computing global cognitive ability while pooling data across different studies, such as composite scores based on the mean of all standardized cognitive scores (Anda et al., 2019). While composite scores, which include all available data and are closer to original test scores, are better able to capture specific deficits and thus more relevant for clinical assessment and treatment, the reductionist approach of PCA simplifies cognitive data by eliminating redundancy and heterogeneity that may hinder between-group comparisons. Moreover, despite the g-factor being an abstract construct that may not directly correspond to specific cognitive processes, it is supported by a robust theoretical background and empirical evidence; conversely, composite scores lack a theoretical foundation, limiting their interpretability and comparisons across studies. Future research on the determinants of cognitive impairment should include the g-factor in larger samples and causal analyses, and investigate subgroups with distinct cognitive trajectories to address the heterogeneity among SZ comprehensively.

## Conclusion

The g-factors derived from diverse cognitive test batteries proved to be valid global cognitive scores across the schizophrenia spectrum, effectively distinguishing between different disease stages on average. The g-factors were used to investigate underlying mechanisms of cognitive impairment within the schizophrenia spectrum. Educational attainment and genetics related to cognitive performance were associated with better cognition, whereas general psychopathology of schizophrenia, neurodevelopmental load, antipsychotic medication, and genetic risk for schizophrenia were linked to poorer cognition. The progressive nature of cognitive deficits across the schizophrenia spectrum, coupled with the positive correlation with educational attainment, provide compelling arguments in favor of early cognitive training interventions to mitigate cognitive decline associated with schizophrenia, and thus prevent the alteration of functional outcomes.

## Supporting information

Yeh et al. supplementary material Yeh et al. supplementary material

## Supplementary material

The supplementary material for this article can be found at https://doi.org/10.1017/S0033291724002538
