# Supplementary material for: Cognitive impairment in the schizophrenia spectrum: exploring the relationships of the g-factor with sociodemography, psychopathology, neurodevelopment, and genetics
Source: Psychol Med. 2024 Dec 2;54(15):4243–54. doi: 10.1017/S0033291724002538 (PMC11650192; doi:10.1017/S0033291724002538)
Supplement: Yeh et al. supplementary material [file S0033291724002538sup001.pdf]

Supplementary Material

Supplementary Table S1. Summary statistics for demographic data, cognitive test scores and psychopathological data, by diagnostic group.

	[ALL] (N = 892)	HC (N = 124)		FDR (N = 163)		UHR (N = 338)		FEP (N = 100)		SZ (N = 167)		Overall group differences (p-value)	Significant pairwise differences
	n	n	mean (SD)	n	mean (SD)	n	mean (SD)	n	mean (SD)	n	mean (SD)		
**Sociodemography**													
Sex	892	124		163		338		100		167			
F – Number (%)			71 (57.3%)		78 (47.9%)		121 (35.8%)		29 (29.0%)		45 (26.9%)		
H – Number (%)			53 (42.7%)		85 (52.1%)		217 (64.2%)		71 (71.0%)		122 (73.1%)		
Age	892	124	24.8 (6.19)	163	52.7 (17.3)	338	20.8 (3.41)	100	24.4 (6.62)	167	29.0 (9.29)	**<0.001**	HC<FDR<br>HC>UHR<br>HC<SZ<br>FDR>UHR<br>FDR>FEP<br>FDR>SZ<br>UHR<FEP<br>UHR<SZ<br>FEP<SZ
Number of years of education	851	118	14.2 (2.45)	143	12.8 (3.80)	335	12.3 (2.67)	98	13.0 (2.45)	157	12.1 (2.77)	**<0.001**	HC<FDR<br>HC<UHR<br>HC<FEP<br>HC<SZ
**WAIS global scores**													
Full-scale IQ (FSIQ)	372	32	109 (12.3)	0	—	251	102 (14.9)	34	96.9 (13.1)	55	97.4 (14.9)	**0.002**	HC>FEP<br>HC>SZ
Performance IQ (PIQ)	323	25	106 (12.9)	0	—	209	97.6 (15.2)	34	92.6 (15.5)	55	92.9 (14.3)	**0.002**	HC>UHR<br>HC>FEP<br>HC>SZ
Verbal IQ (VIQ)	321	25	108 (13.0)	0	—	207	104 (15.1)	34	100 (12.1)	55	100 (14.8)	0.078	—
**WAIS indices (age-scaled scores)**													
Perceptual Reasoning Index (PRI)	277	32	105 (12.5)	0	—	164	102 (13.8)	35	95.1 (15.8)	46	98.4 (13.7)	**0.025**	HC>FEP<br>UHR>FEP
Processing Speed Index (PSI)	51	7	109 (18.5)	0	—	44	101 (14.4)	0	—	0	—	0.187	—
Working Memory Index (WMI)	150	26	108 (16.4)	0	—	81	102 (15.6)	4	96.0 (9.52)	39	92.8 (13.0)	**0.004**	HC>SZ<br>UHR>SZ
Verbal Comprehension Index (VCI)	145	32	111 (14.1)	0	—	66	113 (14.5)	0	—	47	106 (14.7)	**0.030**	UHR>SZ
**WAIS subtests (age-scaled scores)**													
Block Design	367	32	10.7 (3.01)	0	—	243	10.0 (3.49)	35	8.83 (3.56)	57	9.37 (3.48)	0.134	—
Matrix Reasoning	367	32	10.9 (1.77)	0	—	243	10.1 (2.76)	35	9.06 (3.34)	57	10.1 (2.94)	0.104	—
Picture Completion (WAIS-III)	312	25	11.6 (2.20)	0	—	199	10.8 (2.86)	35	9.89 (2.46)	53	9.68 (2.91)	**0.037**	HC>SZ
Visual Puzzles (WAIS-IV)	51	7	9.71 (3.59)	0	—	44	9.66 (3.03)	0	—	0	—	0.996	—
Digit Span	366	32	11.3 (3.59)	0	—	243	9.90 (3.20)	35	8.71 (2.72)	56	8.88 (2.74)	**0.006**	HC>FEP<br>HC>SZ
Arithmetic	364	32	10.8 (2.68)	0	—	243	9.92 (2.95)	35	8.80 (2.84)	54	9.15 (3.67)	0.060	—
Symbol Search (WAIS-IV)	51	7	10.9 (4.38)	0	—	44	10.9 (2.55)	0	—	0	—	0.996	—

Coding	367	32	10.9 (2.50)	0	—	243	8.79 (3.21)	35	7.71 (2.91)	57	7.09 (2.65)	**<0.001**	HC>UHR<br>HC>FEP<br>HC>SZ<br>UHR>SZ
Vocabulary	367	32	12.3 (2.99)	0	—	243	12.0 (3.13)	35	11.3 (2.32)	57	11.2 (3.05)	0.188	—
Information	366	32	11.4 (2.79)	0	—	243	11.1 (3.15)	35	11.3 (2.93)	56	10.9 (3.04)	0.996	—
Similarities	367	32	12.4 (2.56)	0	—	243	11.5 (3.03)	35	10.3 (3.24)	57	11.6 (3.23)	0.095	—
**Minicog**													
Similarities *(age-scaled score)*	367	32	12.4 (2.56)	0	—	243	11.5 (3.03)	35	10.3 (3.24)	57	11.6 (3.23)	0.055	—
Phonemic Verbal Fluency – P letter *(Z-score)*	829	117	0.29 (0.94)	139	-0.11 (1.04)	323	0.41 (0.92)	94	0.08 (1.03)	156	-0.33 (1.01)	**<0.001**	HC>SZ<br>FDR<UHR<br>UHR>FEP<br>UHR>SZ<br>FEP>SZ
Phonemic Verbal Fluency – R letter *(Z-score)*	830	117	0.06 (0.83)	140	-0.07 (0.90)	323	0.28 (0.75)	94	0.07 (0.82)	156	-0.20 (0.87)	**<0.001**	FDR<UHR<br>UHR>SZ
Semantic Verbal Fluency – Animal category *(Z-score)*	828	117	-0.24 (0.78)	139	-0.52 (1.01)	323	-0.44 (0.69)	93	-0.78 (0.77)	156	-1.03 (0.83)	**<0.001**	HC>FDR<br>HC>FEP<br>HC>SZ<br>FDR>SZ<br>UHR>FEP<br>UHR>SZ
Semantic Verbal Fluency – Fruits category *(Z-score)*	827	117	0.46 (1.45)	138	0.05 (1.08)	323	0.35 (1.12)	93	-0.23 (1.21)	156	-0.74 (1.42)	**<0.001**	HC>FEP<br>HC>SZ<br>FDR>SZ<br>UHR>FEP<br>UHR>SZ<br>FEP>SZ
Verbal Fluency – Total *(Z-score)*	829	117	0.15 (0.82)	139	-0.17 (0.77)	323	0.15 (0.64)	94	-0.22 (0.78)	156	-0.57 (0.83)	**<0.001**	HC>FDR<br>HC>FEP<br>HC>SZ<br>FDR<UHR<br>FDR>SZ<br>UHR>FEP<br>UHR>SZ<br>FEP>SZ
Trail Making Test A *(Z-score)*	864	122	0.07 (1.05)	151	-1.02 (1.85)	330	-1.13 (1.90)	99	-1.61 (2.45)	162	-1.79 (2.46)	**<0.001**	HC>FDR<br>HC>UHR<br>HC>FEP<br>HC>SZ<br>FDR>SZ<br>UHR>SZ
Trail Making Test B *(Z-score)*	861	122	-0.37 (1.61)	150	-2.39 (6.79)	330	-1.78 (2.83)	99	-2.80 (2.96)	160	-4.29 (5.66)	**<0.001**	HC>FDR<br>HC>UHR<br>HC>FEP<br>HC>SZ<br>FDR>SZ<br>UHR>SZ
Trail Making Test B–A *(Z-score)*	870	122	-0.33 (1.34)	159	-1.46 (5.72)	330	-1.09 (2.52)	99	-1.85 (2.88)	160	-3.34 (5.26)	**<0.001**	HC>FEP<br>HC>SZ<br>FDR>SZ<br>UHR>SZ
Proverbs (PANSS N5 item)	491	43	2.51 (1.01)	99	2.24 (1.41)	169	1.58 (0.97)	81	2.89 (1.32)	99	3.47 (1.53)	**<0.001**	HC>UHR<br>HC<SZ<br>FDR>UHR<br>FDR<FEP<br>FDR>SZ<br>UHR<FEP<br>UHR<SZ<br>FEP<SZ

fNART – Total IQ	274	30	108 (5.17)	0	—	158	107 (6.61)	35	106 (6.42)	51	106 (8.25)	0.484	—
fNART – Performance IQ	274	30	105 (2.89)	0	—	158	105 (3.87)	35	104 (4.49)	51	104 (4.53)	0.453	—
fNART – Verbal IQ	274	30	110 (6.52)	0	—	158	108 (8.38)	35	107 (8.24)	51	107 (10.4)	0.519	—
Coupled Words	273	22	26.5 (4.51)	0	—	120	24.7 (5.85)	74	17.7 (7.09)	57	19.6 (6.79)	**<0.001**	HC>FEP<br>HC>SZ<br>UHR>FEP<br>UHR>SZ
**Psychopathology**													
PANSS – Positive scale	433	0	—	24	14.0 (5.60)	169	14.3 (4.67)	96	22.9 (7.58)	143	16.9 (6.80)	**<0.001**	FDR<FEP<br>UHR<FEP<br>UHR<SZ<br>FEP>SZ
PANSS – Negative scale	433	0	—	24	20.9 (9.66)	169	15.7 (6.61)	96	23.6 (7.53)	143	23.1 (8.38)	**<0.001**	FDR>UHR<br>UHR<FEP<br>UHR<SZ
PANSS – General Psychopathology scale	433	0	—	24	34.8 (15.3)	169	13.9 (14.2)	96	32.0 (19.0)	143	40.2 (12.6)	**<0.001**	FDR>UHR<br>UHR<FEP<br>UHR<SZ<br>FEP<SZ
PANSS – Total	433	0	—	24	69.8 (27.6)	169	43.8 (16.7)	96	78.4 (23.8)	143	80.3 (24.3)	**<0.001**	FDR>UHR<br>UHR<FEP<br>UHR<SZ
BPRS – Total	734	45	25.9 (3.28)	145	31.8 (11.6)	288	56.1 (14.4)	97	68.6 (15.9)	159	56.2 (17.1)	**<0.001**	HC<UHR<br>HC<FEP<br>HC<SZ<br>FDR<UHR<br>FDR<FEP<br>FDR<SZ<br>UHR<FEP<br>FEP>SZ
**Neurodevelopmental load**													
DDSS – Social Relationships	173	8	0.25 (0.71)	0	—	104	1.21 (1.87)	32	1.12 (1.68)	29	1.41 (1.55)	0.446	—
DDSS – Communication	174	7	0.43 (0.79)	0	—	108	0.63 (0.93)	30	0.63 (1.00)	29	0.59 (0.82)	0.950	—
DDSS – Behaviour	175	8	0.25 (0.46)	0	—	107	0.62 (0.91)	31	0.26 (0.63)	29	0.69 (0.97)	0.165	—
DDSS – Global Development	176	8	1.00 (1.77)	0	—	107	1.42 (1.59)	32	1.72 (2.29)	29	2.14 (1.96)	0.265	—
DDSS – Total	170	7	2.14 (3.24)	0	—	102	3.74 (3.70)	32	3.69 (3.97)	29	4.83 (3.74)	0.353	—
NSS – Motor Coordination factor	615	111	3.70 (3.03)	144	6.24 (4.34)	120	3.58 (2.52)	95	5.61 (3.39)	145	7.62 (5.18)	**<0.001**	HC<FDR<br>HC<FEP<br>HC<SZ<br>FDR>UHR<br>FDR<SZ<br>UHR<FEP<br>UHR<SZ<br>FEP<SZ
NSS – Motor Integration factor	615	110	0.62 (0.96)	145	1.93 (2.13)	120	0.94 (1.22)	95	1.90 (2.09)	145	2.87 (2.72)	**<0.001**	HC<FDR<br>HC<FEP<br>HC<SZ<br>FDR>UHR<br>FDR<SZ<br>UHR<FEP<br>UHR<SZ<br>FEP<SZ

NSS – Sensory Integration factor	617	112	1.13 (1.19)	145	2.77 (2.41)	120	1.22 (1.48)	95	2.09 (1.90)	145	3.38 (2.63)	**<0.001**	HC<FDR<br>HC<FEP<br>HC<SZ<br>FDR>UHR<br>FDR<SZ<br>UHR<FEP<br>UHR<SZ<br>FEP<SZ
NSS – Involuntary Movements factor	613	111	0.42 (0.74)	140	0.56 (0.95)	120	0.12 (0.41)	95	0.38 (0.86)	147	0.83 (1.33)	**<0.001**	HC<SZ<br>FDR>UHR<br>UHR<SZ<br>FEP<SZ
NSS – Lateralisation factor	616	111	0.62 (1.07)	143	0.67 (1.07)	120	0.86 (1.18)	95	1.06 (1.44)	147	0.93 (1.33)	0.062	—
NSS – Total	618	112	6.30 (4.40)	146	12.0 (8.08)	120	6.16 (3.33)	95	10.8 (6.15)	145	15.5 (10.6)	**<0.001**	HC<FDR<br>HC<FEP<br>HC<SZ<br>FDR>UHR<br>FDR<SZ<br>UHR<FEP<br>UHR<SZ<br>FEP<SZ
**Medication**													
Chlorpromazine equivalent dose (mg)	256	0	–	0	–	172	21.1 (55.5)	37	119 (195)	47	255 (186)	**<0.001**	UHR<FEP<br>UHR<FEP<br>FEP<SZ

n: number of individuals with non-missing values; SD: standard deviation; age-scaled score: scaled score according to the norms by age provided by the WAIS-III or WAIS-IV Administration and Scoring Manual (Wechsler, 1997;2008); Z-score: score standardised by age and/or sex and/or number of years of education, according to the respective norms for each test (Verbal Fluency Test: Cardebat, 1990 / TMT: Tombaugh, 2004).

HC: healthy controls; FDR: first-degree family members of patients; UHR: patients at ultra-high risk of psychosis; FEP: patients who had a first episode of psychosis; SZ: patients with schizophrenia.

PANSS: Positive And Negative Symptoms Scale; BPRS: Brief Psychiatric Rating Scale; DDSS: Developmental Disorders Screening Scale; NSS: Neurological Soft Signs Examination.

The descriptives and tests are performed using the R package compareGroups *(Subirana et al., 2014). Variables are compared using ANOVA tests, which* p*-values are reported in the table. When the results of the ANOVA are significant, post-hoc tests are performed for pairwise comparisons corrected using Benjamini-Hochberg method.*

Supplementary Table S2. Significant correlations of the g-factors with sociodemographic and psychopathological data.

	g WAIS global	g WAIS III	g WAIS 8	g WAIS 2	g Minicog 6	g Minicog 3	g Mix
**Sociodemography**							
Age						r=-0.13, p=0.013	
Number of years of education	r=0.45, p<.001	r=0.44, p<.001	r=0.44, p<.001	r=0.44, p<.001	r=0.44, p<.001	r=0.28, p<.001	r=0.42, p<.001
**Psychopathology – PANSS**							
PANSS – Positive scale			r=-0.16, p=0.032			r=-0.32, p=<.001	r=-0.20, p=0.007
PANSS – Negative scale					r=-0.30, p=<.001	r=-0.51, p=<.001	r=-0.33, p=<.001
PANSS – General Psychopathology scale	r=-0.22, p=0.004	r=-0.22, p=0.005		r=-0.21, p=0.003	r=-0.43, p=<.001	r=-0.46, p=<.001	r=-0.30, p=<.001
PANSS – Total				r=-0.19, p=0.006	r=-0.4, p=<.001	r=-0.55, p=<.001	r=-0.35, p=<.001
**Psychopathology – BPRS**							
BPRS – Total						r=-0.19, p=0.001	
**Neurodevelopmental load – NSS**							
NSS – Motor Coordination factor						r=-0.31, p=<.001	
NSS – Motor Integration factor	r=-0.20, p=0.008	r=-0.18, p=0.022	r=-0.17, p=0.031		r=-0.19, p=0.027	r=-0.36, p=<.001	r=-0.18, p=0.027
NSS – Sensory Integration factor	r=-0.32, p=<.001	r=-0.33, p=<.001	r=-0.33, p=<.001	r=-0.26, p=<.001	r=-0.25, p=0.002	r=-0.36, p=<.001	r=-0.29, p=<.001
NSS – Involuntary Movements factor						r=-0.20, p=<.001	
NSS – Total	r=-0.18, p=0.019	r=-0.18, p=0.022	r=-0.17, p=0.026	r=-0.18, p=0.016		r=-0.38, p=<.001	r=-0.23, p=0.003
**Medication**							
Chlorpromazine equivalent dose	r=-0.24, p=0.002	r=-0.23, p=0.004	r=-0.24, p=<.001	r=-0.19, p=0.009	r=-0.31, p=<.001	r=-0.40, p=<.001	r=-0.34, p=<.001

r: Spearman's correlation; p: adjusted p-*value corrected for multiple comparisons with Benjamini-Hochberg method.*

PANSS: Positive And Negative Symptoms Scale; BPRS: Brief Psychiatric Rating Scale; DDSS: Developmental Disorders Screening Scale; NSS: Neurological Soft Signs Examination.

Supplementary Table S3. Linear regressions of the g-factors against the PRS and meta-analyses.

g-factor	Linear regressions of the g-factors against the PRS						Meta-analyses	
	PRS	P_T	β	SE	PRS.R2	P	COR	W
g Minicog 3	Cognitive performance	1e-5	0.0780	0.0744	0.005	0.2955	0.0778	18.6
	Schizophrenia	1e-5	-0.1503	0.0725	0.021	**0.0398**	-0.1499	18.4
g Minicog 6	Cognitive performance	1e-5	0.2490	0.0981	0.050	**0.0128**	0.2479	11.1
	Schizophrenia	1e-5	-0.2040	0.0947	0.037	**0.0337**	-0.2031	11.2
g Mix	Cognitive performance	1e-4	-0.0443	0.0896	0.002	0.622	-0.0441	15.1
	Schizophrenia	1e-5	0.0615	0.0878	0.003	0.485	0.0613	15.0
g WAIS 2	Cognitive performance	1e-4	-0.0443	0.0896	0.002	0.622	-0.0441	15.1
	Schizophrenia	1e-5	0.0615	0.0878	0.003	0.485	0.0613	15.0
g WAIS 8	Cognitive performance	0.3	0.1850	0.0874	0.029	**0.0362**	0.1844	14.8
	Schizophrenia	0.2	-0.0381	0.0918	0.001	0.6786	-0.0380	15.0
g WAIS global	Cognitive performance	0.3	0.1719	0.0919	0.025	0.0639	0.1713	13.6
	Schizophrenia	0.1	-0.0728	0.0956	0.004	0.4479	-0.0725	13.7
g WAIS-III	Cognitive performance	0.3	0.1430	0.0922	0.018	0.1238	0.1425	13.5
	Schizophrenia	1e-6	-0.0449	0.0917	0.002	0.625	-0.0447	13.6

PRS: polygenic risk score; P_T: most predictive p-value threshold for the association between SNPs and the GWAS phenotype; β: standardised estimate of the regression; SE: standard error of the estimate; PRS.R2: proportion of g-factor variance explained by the PRS; P: p-value of the association between the g-factor and the PRS; COR: effect size as correlation (conversion from β); W: weight (in percent) attributed to each study in the meta-analyses.

In bold: statistically significant results ($p < 0.05$).

The linear regressions are performed at the best-fit p-value threshold for each GWAS, and adjusting for age, sex, diagnostic status, and population stratification (ten principal components).

Supplementary Table S4. Meta-analysis of the results of the regressions of the g-factors on the PRS, based on the generic inverse variance method with a fixed-effects model.

PRS	r_{pooled}	I^2	P
Cognitive performance	0.1138	58.7%	**< 0.0001**
Schizophrenia	-0.0810	51.8%	**0.0004**

PRS: polygenic risk score; r_{pooled}*: pooled effect size;* I^2*: Higgins and Thompson's between-sample heterogeneity index; P:* p-*value for the effect size.*

In bold: statistically significant results ($p < 0.05$ *).*
